# Implementation of the Adductor Strengthening Programme: Players primed for adoption but reluctant to maintain — A cross‐sectional study

**DOI:** 10.1111/sms.13444

**Published:** 2019-05-23

**Authors:** Joar Harøy, Espen Guldahl Wiger, Roald Bahr, Thor Einar Andersen

**Affiliations:** ^1^ Department of Sports Medicine, Oslo Sports Trauma Research Center Norwegian School of Sport Sciences Oslo Norway

**Keywords:** attitudes, football, groin injury, implementation, injury prevention

## Abstract

Groin injuries represent a considerable problem in male football, accounting for 4%‐19% of all time‐loss injuries. The Adductor Strengthening Programme is the first groin‐specific prevention program shown to reduce the risk of groin problems. We aimed to use the RE‐AIM framework to examine the players’ experiences with the implementation of the program and player attitude toward groin injury prevention in football. Of the 632 players involved in the trial examining the effect of the Adductor Strengthening Programme, 501 agreed to participate in a survey at the end of the season. Most players thought that footballers are at moderate to high risk for groin injuries (87%) and that there is a need for preventive measures (96%). They also believed that a preventive program with strengthening exercises would reduce the risk of groin injuries (91%). Majority of the players reported using <5 minutes to complete the program (73%), and only 11% wanted additional exercises. However, only 46% reported to have performed the program as recommended, and an even smaller proportion (31%) planned to continue using it as recommended the next season. Our results suggest that footballers believe that prevention of groin injuries is needed. Attitude toward implementation of the Adductor Strengthening Programme was positive, and the single‐exercise approach was considered an important facilitator. However, in future dissemination of the program, the players’ reluctance to maintain the exercise protocol may be a potential barrier to implementation that should be addressed.

## 1 INTRODUCTION

Groin injuries represent a considerable problem in male football, accounting for 4%‐19% of all time‐loss injuries.^1^ At the elite level, 14%‐17% of all players incur a groin injury causing time loss each season.^2^ During a period with match congestion, 59% of males reported at least one episode with groin problems.^3^

Several preventive measures have been suggested to reduce the high groin injury rates. Until recently, groin‐specific exercise programs aiming to prevent groin injuries have not shown any significant effects.^4^ The groin‐specific interventions have had a combined focus on hip adductors, flexors, and abdominals.^5^, ^6^ However, recently, the Adductor Strengthening Programme, a simple, single‐exercise program based on the Copenhagen Adduction exercise,^7^ was shown to reduce the risk of groin problems among male football players with 41%.^8^

In this study, the compliance with the Adductor Strengthening Programme was higher than what has previously been reported in groin‐specific injury prevention trials.^4^, ^5^, ^6^ On average, the players completed approximately 70% of the prescribed training sessions both during the pre‐season and the regular season.^8^

Although better compliance to injury prevention program has demonstrated lower injury rates throughout the season,^9^, ^10^, ^11^ it is well known that compliance with prevention programs represents a challenge.^12^, ^13^, ^14^, ^15^ From elite European level, it has been shown that players compliance with prevention programs is low, despite coaches being positive.^14^ Poor compliance with prevention programs with documented effect may be considered as a potential barrier to implementation, indicating a gap between science and real‐world implementation. The RE‐AIM framework has been developed to describe five key components to successfully close the gap: reach, efficacy, adoption, implementation, and maintenance.^16^, ^17^ The framework is a useful tool that allows decision‐makers to assess how interventions are implemented in practice, and their impact at the individual and organizational levels. Furthermore, it can help determine which interventions are feasible in real‐world settings.^16^, ^17^, ^18^ A recent systematic review concluded that information on the RE‐AIM components in published trials on injury prevention exercise programs was insufficient, especially regarding the adoption and maintenance of the programs.^16^ By using the RE‐AIM framework to evaluate the implementation of the Adductor Strengthening Programme, we may reveal factors important for future successful dissemination.

Thus, the primary aim of this study was to use the RE‐AIM framework to examine the players’ experiences with the implementation of the Adductor Strengthening Programme among male sub‐elite football players. Furthermore, we investigated player attitudes toward groin injury prevention.

## 2 METHODS

### 2.1 Study design and participants

This survey took place from September to October 2016, as part of a randomized controlled trial (RCT) examining the preventive effect of the Adductor Strengthening Programme on the risk of groin problems in sub‐elite male football players.^8^ The RCT was registered with the International Standard Randomised Controlled Trial Number registry (ISTRCTN98514933); however, the present data collection was not pre‐planned or registered as a part of the protocol. The South‐Eastern Norway Regional Committee for Medical Research Ethics approved the changes (2015/1922/REK).

During the last weeks of the 2016 season, we invited all 34 teams (632 players) enrolled in the RCT to take part in the study. Teams that agreed to participate received a personal visit, where we informed players about the study, and each player was invited to participate. All players enrolled in the RCT were eligible for participation. All players received oral and written information about the purposes and procedures of the project before providing their written consent.

This report is prepared according to the STROBE checklist for observational studies.^19^

### 2.2 Questionnaire

The survey was based on the different dimensions of the RE‐AIM framework^18^ and was developed based on a similar survey used to examine the implementation of the OSTRC Shoulder Injury Prevention Programme in handball players.^20^ We included a separate section with questions about the implementation of the Adductor Strengthening Programme for players in the intervention group and questions regarding knowledge about the program for players in the control group. In addition, we included questions about attitudes toward groin injuries and groin injury prevention for both groups. The survey was developed in Norwegian, and an English version was provided for players who did not understand Norwegian. The full survey is available as an Appendix S1.

### 2.3 Procedure

Players were asked to complete a paper version of the questionnaire. Players who were injured, ill, or for other reasons did not attend training the day of our personal visit were contacted through e‐mail or phone and asked to complete an online version of the questionnaire using Questback (Questback V. 9692, Questback AS).

### 2.4 Analysis

All returned questionnaires were included in the analysis regardless of missing data. All responses were exported into SPSS Statistics for Windows, version 24.0 (SPSS Inc), and analyzed using descriptive statistics. Player characteristics were obtained from the RCT (at the time of inclusion in February and March 2016).^8^ All data are presented as mean and standard deviation (SD).

## 3 RESULTS

### 3.1 Participant characteristics

Three of the 34 teams in the RCT declined to participate. In total, 501 (79%) of the players enrolled at baseline were included, 75% from the intervention group (n = 255), and 84% from the control group (n = 246). Of the 501, 408 (81%) responded to the questionnaire during our team visit, while 93 responded to the online version of the questionnaire within 2 weeks. Player characteristics at baseline are shown in Table 1.

**Table 1:** Baseline characteristics for players included in the study. Values are expressed as mean (SD)

	Intervention (n = 255)	Control (n = 246)
Age (y)	22.0 (4.4)	23.8 (4.4)
Height (cm)	181.9 (6.5)	182.4 (6.4)
Body mass (kg)	75.8 (7.5)	78.1 (7.5)
Senior player (y)^a^	5.4 (4.1)	6.3 (4.5)

a Years playing senior football.

### 3.2 Player attitudes toward groin injuries and groin injury prevention

Overview of the player responses regarding their attitude toward groin injuries and groin injury prevention is shown in Tables 2 and 3. Of the players included in the intervention, 87.3% considered footballers to be exposed to groin injuries to a moderate or high extent. The players perceived low muscle strength (23.0%), reduced mobility (21.1%), and playing on artificial turf (18.4%) as the most important causes of groin injuries. Furthermore, prevention of groin injuries was considered at least moderately important by 95.5% of the players. However, when asked about their squad’s attitude toward preventive measures, their impression was that only 50.1% of the players were positive.

**Table 2:** Questions and responses from players about their attitude to groin injuries and groin injury prevention. Data are presented as number of players (%)

	Intervention (n = 255)	Control (n = 246)	Total (n = 501)
To what extent do you think footballers are exposed to groin injuries?			
Highly	114 (44.7)	123 (50.0)	237 (47.3)
Moderately	107 (42.0)	94 (38.2)	201 (40.1)
Low	24 (9.4)	20 (8.1)	44 (8.8)
Don’t know	4 (1.6)	6 (2.4)	10 (2.0)
Missing	6 (2.4)	3 (1.2)	9 (1.8)
To what extent do you think footballers need to prevent groin injuries?			
Highly	159 (62.4)	160 (65.0)	319 (63.7)
Moderately	84 (33.0)	75 (30.5)	159 (31.8)
Low	8 (3.1)	8 (3.3)	16 (3.2)
Don’t know	1 (0.4)	3 (1.2)	4 (0.8)
Missing	3 (1.2)	0 (0.0)	3 (0.6)
What do you think are the most common causes of groin injuries among footballers?^a^			
Too little training	57 (4.6)	61 (4.8)	118 (4.7)
Too much training	108 (8.7)	157 (12.5)	265 (10.6)
Too many matches	68 (5.5)	67 (5.3)	135 (5.4)
Hard tackles	2 (0.2)	4 (0.3)	6 (0.2)
Low muscle strength	302 (24.2)	275 (21.8)	577 (23.0)
Reduced mobility	273 (21.9)	256 (20.3)	529 (21.1)
Reduced recovery time between matches	96 (7.7)	91 (7.2)	187 (7.5)
Artificial turf	212 (17.0)	250 (19.9)	462 (18.4)
Other	43 (3.5)	51 (4.0)	94 (3.8)
Missing	86 (6.9)	48 (3.8)	134 (5.4)
It is more important to use the training time to play football than to conduct injury prevention			
Fully agree	58 (22.7)	38 (15.4)	96 (19.2)
Agree	125 (49.0)	95 (38.6)	220 (43.9)
Not sure	32 (12.5)	43 (17.5)	75 (15.0)
Disagree	11 (4.3)	56 (22.8)	67 (13.4)
Totally disagree	2 (0.8)	8 (3.3)	10 (2.0)
Don’t know	19 (7.5)	3 (1.2)	22 (4.4)
Missing	8 (3.1)	3 (1.2)	11 (2.2)
The motivation of the coach affects the players motivation to conduct prevention exercises			
Fully agree	58 (22.7)	50 (20.3)	108 (21.5)
Agree	125 (49.0)	133 (54.0)	258 (51.5)
Not sure	32 (12.5)	43 (17.5)	75 (15.0)
Disagree	11 (4.3)	11 (4.5)	22 (4.4)
Totally disagree	2 (0.8)	2 (0.8)	4 (0.8)
Don’t know	19 (7.5)	4 (1.6)	23 (4.6)
Missing	8 (3.1)	3 (1.2)	11 (2.2)

Note

Total number of answers: Intervention n = 1247; control n = 1260; total n = 2507.

a Multiple responses possible.

**Table 3:** Question and response from players regarding attitude of prevention measures in different staffs of the club. Data are presented as number of players (%)

	Very positive	Positive	Neutral	Negative	Very negative	Don’t know	Missing
How do you perceive the general attitude to preventive measures in the following group in your club?							
Intervention (n = 255)							
Coaches	73 (28.6)	112 (43.9)	50 (19.6)	1 (0.4)	0 (0.0)	11 (4.3)	8 (3.1)
Medical teams	90 (35.3)	101 (39.6)	28 (11.0)	1 (0.4)	0 (0.0)	27 (10.6)	8 (3.1)
Players	40 (15.7)	96 (37.6)	90 (35.3)	11 (4.3)	0 (0.0)	9 (3.5)	9 (3.5)
Administration	32 (12.5)	39 (15.3)	60 (23.5)	1 (0.4)	2 (0.8)	112 (43.9)	9 (3.5)
Control (n = 246)							
Coaches	54 (22.0)	112 (45.5)	57 (23.1)	14 (5.7)	3 (1.2)	3 (1.2)	3 (1.2)
Medical teams	85 (34.5)	100 (40.6)	39 (15.9)	6 (2.4)	2 (0.8)	11 (4.5)	3 (1.2)
Players	30 (12.2)	85 (34.5)	107 (43.5)	16 (6.5)	2 (0.8)	3 (1.2)	3 (1.2)
Administration	21 (8.5)	45 (18.3)	92 (37.4)	12 (4.9)	5 (2.0)	68 (27.7)	3 (1.2)
Total (n = 501)							
Coaches	127 (25.4)	224 (44.7)	107 (21.4)	15 (3.0)	3 (0.6)	14 (2.8)	11 (2.2)
Medical teams	175 (35.0)	201 (40.1)	67 (13.4)	7 (1.4)	2 (0.4)	38 (7.6)	11 (2.2)
Players	70 (14.0)	181 (36.1)	197 (39.2)	27 (5.4)	2 (0.4)	12 (2.4)	12 (2.4)
Administration	53 (10.6)	84 (16.8)	152 (30.3)	13 (2.6)	7 (1.4)	180 (36.0)	12 (2.4)

### 3.3 Player experience with the Adductor Strengthening Programme

The questions and responses from players in the intervention group to the questions about the Adductor Strengthening Programme are shown in Tables 4 and 5. Strengthening of the adductor muscles was considered important in reducing groin problems by 90.6% of the players in the intervention group. Of the players in the intervention group, 72.5% reported that they spent <5 minutes to complete the program. Two‐thirds (66.7%) of the players reported that they appreciated that the program consisted of a single exercise and believed motivation for doing the program would decrease if it were more time‐consuming. Only 11.4% of the players wanted additional exercises. Sixty‐six percent of the players reported that the program was performed in connection with organized football training. Most players (52.0%) reported that coaches were responsible for initiating the Adductor Strengthening Programme during training, while other players (36.8%) reported that players themselves initiated the program. Coaches (40.7%) and players (46.8%) were also responsible for the players performing the exercises with the same quality as instructed. The exercise protocol was conducted as recommended or more frequently than recommended by 45.9% of the players. When players were asked whether they thought they would perform the program the following season, 64.7% of the players confirmed they would; however, of these, 52.2% reported that they would do the program less frequently.

**Table 4:** Questions and response from players from the intervention group (n = 255) about the Adductor Strengthening Programme

	Number of players (%)
Are you familiar with the Adductor Strengthening Programme intended to prevent groin injuries?	
Yes	242 (94.9)
No	6 (2.4)
Don’t know	2 (0.8)
Missing	5 (2.0)
Do you believe that the Adductor Strengthening Programme can reduce groin injuries?	
Yes, definitely	137 (53.7)
Yes, somewhat	94 (36.9)
No, I don’t think so	6 (2.4)
Don’t know	13 (5.0)
Missing	5 (2.0)
Which players have primarily conducted the program?	
All or most players	134 (52.5)
Players with groin problems	30 (11.8)
Players with previous groin problems	21 (8.2)
No players	7 (2.7)
Don’t know	56 (22)
Missing	7 (2.7)
How has the execution of the program been organized?	
When the players wanted, but not connected to organized training	68 (26.7)
When the players wanted, but connected to organized training (before or after training)	55 (21.6)
Together as a team connected to organized training	114 (44.7)
Don’t know	9 (3.5)
Missing	9 (3.5)
Have you conducted the program with the recommended frequency?	
More often	16 (6.3)
As recommended	101 (39.6)
Less often	114 (44.7)
Don’t know	18 (7.0)
Missing	6 (2.4)
How much time did you spend conducting the Adductor Strengthening Program?	
0‐5 min	185 (72.5)
5‐10 min	53 (20.8)
10‐15 min	3 (1.2)
Don’t know	9 (3.5)
Missing	5 (2.0)
Will you use the program after the current season?	
Yes, definitely	79 (31.0)
Yes, but not as frequently as this season	86 (33.7)
No	49 (19.2)
Don’t know	40 (15.7)
Missing	1 (0.4)
Do you think the motivation to perform the Adductor Strengthening Program would have been greater if the exercise was not a partner exercise?	
Yes, it is better to train alone	35 (13.7)
It does not matter	119 (46.7)
No, it is better to train with a team‐mate	77 (30.2)
Don’t know	20 (7.8)
Missing	4 (1.6)
Do you think the motivation to perform the program would have been greater if it had contained several exercises?	
Yes, the more exercises the better	29 (11.4)
No, one exercise is sufficient	170 (66.7)
Don’t know	51 (20.0)
Missing	5 (2.0)
How would the motivation to perform the program have changed if it had taken less time?	
It would have increased	90 (35.3)
It would have decreased	7 (2.7)
Don’t know	152 (59.6)
Missing	6 (2.4)
How would the motivation to perform the program have changed if it had taken more time?	
It would have increased	20 (7.8)
It would have decreased	151 (59.2)
Don’t know	80 (31.4)
Missing	4 (1.6)

**Table 5:** Questions and response from players from the intervention group (n = 255) about the implementation of the Adductor Strengthening Programme

**Are the following staff members familiar with the Adductor Strengthening Programme?**	**Yes**	**No**	**Don’t know**	**Missing response**	
Head and assistant coach	219 (85.9)	3 (1.2)	27 (10.6)	6 (2.4)	
Medical team	202 (79.2)	9 (3.5)	36 (14.1)	8 (3.1)	
Other coaches (fitness coach, goalkeeper trainer etc)	180 (70.6)	11 (4.3)	52 (20.4)	12 (4.7)	
**How do you perceive the attitudes to the Adductor Strengthening Programme in the following groups?**	**Positive**	**Neutral**	**Negative**	**Don’t know**	**Missing response**
Head and assistant coach	126 (49.4)	88 (34.5)	4 (1.6)	23 (9.0)	14 (5.5)
Medical team	137 (53.7)	62 (24.3)	3 (1.8)	37 (14.5)	16 (6.3)
Players	63 (24.7)	128 (50.2)	33 (13.0)	15 (5.9)	16 (6.3)
Administration	46 (18.0)	64 (25.1)	3 (1.2)	128 (50.2)	14 (5.5)
**Who has mainly initiated the program? Rate from 1 to 3, where 1 is the one who has initiated it the most.**	**Most**	**Second most**	**Third**		
Head coach	35 (15.7)	39 (23.1)	39 (25.5)		
Assistant coach	3 (1.3)	27 (16.0)	18 (11.8)		
Fitness coach	78 (35.0)	28 (16.6)	13 (8.5)		
Health professional	17 (7.6)	20 (11.8)	12 (7.8)		
Team captain	11 (5.0)	7 (4.1)	14 (9.1)		
Another player of the team	6 (2.7)	26 (15.4)	14 (9.1)		
The players of the team	65 (29.1)	18 (10.7)	19 (12.4)		
Don’t know	8 (3.6)	4 (2.3)	24 (15.7)		
**Who has mainly been responsible for the quality of the Adductor Strengthening Programme? Rate from 1 to 3, where 1 is the one who had most.**	**Most**	**Second most**	**Third**		
Head coach	8 (3.7)	31 (18.4)	47 (31)		
Assistant coach	4 (1.8)	19 (11.2)	19 (12.5)		
Fitness coach	76 (35.2)	31 (18.4)	17 (11.2)		
Health professional	16 (7.4)	27 (16.0)	7 (4.6)		
Team captain	8 (3.7)	9 (5.3)	8 (5.3)		
Another player of the team	4 (1.9)	27 (16.0)	13 (8.6)		
The players of the team	89 (41.2)	19 (11.2)	15 (9.9)		
Don’t know	11 (5.1)	6 (3.6)	26 (17.1)		

### 3.4 Groin injury prevention done by players in the control group

Of the players in the control group, 30.5% reported having knowledge about the content of the Adductor Strengthening Programme and 53.3% reported that they had performed the program or other exercises to prevent groin injuries during the season the study took place.

## 4 DISCUSSION

The aim of this study was to examine the players’ experiences with the implementation of the Adductor Strengthening Programme and to investigate their attitudes toward groin injury prevention. The main findings of this survey of Norwegian semi‐professional football players were that most players believed that footballers are at moderate to high risk for groin injuries and that there is a need for groin injury preventive training or other measures. Most players thought that a preventive program with strengthening exercises would reduce the prevalence of groin injuries. Majority of the players reported using <5 minutes to complete the program, and very few wanted additional exercises. On the other hand, <50% of the players reported to have performed the program as much as recommended during the trial, and an even smaller proportion planned to continue using it as prescribed the next season.

The recently reported preventive effect of the Adductor Strengthening Programme suggests that dissemination and widespread use in the football community would be beneficial.^8^ However, to succeed in a real‐world setting, knowledge regarding attitudes, beliefs, and current behaviors toward groin injury prevention among the delivery agents and football players is crucial, as is identification of facilitators and barriers to implementation.^21^, ^22^ In line with epidemiological data, most players in the present study agreed that footballers are at least moderately exposed to groin problems. Their understanding is in accordance with the literature, which documents that groin injuries are prevalent in football.^1^, ^2^, ^3^ Overall, more than 90% of the players believed that there is a moderate to great need for prevention of groin injuries. This result is line with studies recommending preventive initiatives in football.^1^, ^2^, ^3^, ^4^, ^23^ Furthermore, the surveyed players believed that a program targeting hip adduction strength would reduce the risk of groin problems. Hence, the footballers seemed to be primed for adoption and implementation of the Adductor Strengthening Programme, which is an important premise to succeed with prevention measures.^24^

Despite these results, suggesting that reach of the program was successful, the players reported to deviate from the recommended protocol. In the present study, about 45% of the players reported to have performed the program at least as often as prescribed, while data from the RCT documented an average weekly completion of the program of 70%.^8^ However, the discrepancy may not be contradictory. In the present study, a player may choose to respond *“less often*” to the question *“Have you conducted the program with the recommended frequency?”* if he has missed just one session. Thus, players may have performed the exercise less often and still have 70% compliance. Although player compliance with the program was somewhat lower than our recommendations, the reported compliance in this trial was much higher than that seen in previous groin‐specific prevention trials.^4^, ^5^, ^6^ Compliance is thought to be a key success factor; in two large RCTs testing the effects of the FIFA 11+ program, the risk of sustaining an injury was lower for the high compliance group, compared with players having intermediate or low compliance.^25^, ^26^

The single‐exercise approach should be considered as an important facilitator for the successful implementation of the Adductor Strengthening Programme. Only 11% of the players wanted more exercises. They also believed that a program with several exercises, requiring more time to be spent, would decrease their motivation. The majority of the players reported using <5 minutes to complete the program. In other studies reporting on the uptake of the injury prevention exercise programs in football, the length of the program has been emphasized as one important barrier to implementation.^5^, ^10^ However, a simple exercise program is no guarantee for successful implementation. The preventive effect of the Nordic Hamstring exercise is well known^27^, ^28^, ^29^ and in two RCTs, compliance with the program was 91%.^28^, ^29^ Despite this, elite clubs chose other strategies to prevent hamstring injuries.^12^ Bahr et al speculated that limited influence by the medical team on coaching practices and a lack of focus on injury prevention in education programs targeting coaching staff could be the reason.^12^

A common understanding among the different stakeholders within a club is emphasized as an important premise to succeed with implementation of preventive measures.^21^ Players reported that the coaching staff and players initiated and were responsible for the quality of the Adductor Strengthening Programme, while medical staff members were not much involved. This indicates that coaches and players were the most important facilitators for implementation and compliance with the program, perhaps because the study was done at a sub‐elite level, where medical staff resources are limited. Although 65% of the players planned to perform the program the following season, only 31% planned to perform it according to the prescribed protocol. The reluctant motivation to continue performing the Adductor Strengthening Programme represents a potential barrier to maintenance. This should be addressed when implementing the program in football teams, in particular at lower levels of play where access to medical teams is limited.

Interestingly, 31% of the players in the control group reported to have knowledge about and more than 50% to have performed the Adductor Strengthening Programme or other prevention exercises to reduce the risk of groin injuries during the study period. This suggests that there was a crossover effect in the RCT, suggesting that the 41% reduction in risk of groin problems observed may represent an underestimation.

From elite level, we know that the main challenges with exercise programs to prevent injury are concerns about muscle soreness and “heavy legs.”^14^ Players in the present study were from a lower level, and although we have no data, we would be surprised if they were less concerned about muscle soreness and “heavy legs.” Thus, when introducing the Adductor Strengthening Programme players should be informed that the Copenhagen Adduction exercise, which is the most strenuous level of the program, hardly causes any muscle soreness, when using a similar exercise protocol as in the pre‐season.^30^ Players in the present study were positive to the preventive effect from the Adductor Strengthening Programme, in contrast to reports from elite players, which have shown skepticism toward the effect from preventive programs.^14^ Although players in the present study were positive, in future dissemination of the program, the documented preventive effect of the Adductor Strengthening Programme^8^ should be highlighted as a positive effect on performance. Increased participation of players without groin problems may increase the individual and team performance positively by increasing player availability.^31^, ^32^ Unfortunately, there is no guarantee that increased knowledge about prevention among players automatically will translate into changed behavior, as the learning process and experiences of each individual will affect adoption and implementation of the program.^33^ In order to succeed with behavioral modifications, it is suggested that programs should be introduced from an early age to become an accepted part of their training or warm‐up routine and culture.^33^ Thus, dissemination of prevention exercises and programs should target young players and instructions on how to perform it should be a mandatory part of football coach education at all levels.

### 4.1 Methodological limitation

There are some methodological limitations that should be kept in mind when interpreting the current results. First, this survey included only perceptions of the players. The RE‐AIM framework is a tool for decision‐makers to assess how interventions are implemented in practice, and their impact at the individual and organizational level. The understanding and perceptions of other stakeholders in the club are not known. Furthermore, this study included only teams from the sub‐elite level; we do not know if teams at other levels of play (eg, elite or amateur) or females would have had different perceptions and views.

## 5 PERSPECTIVES

Players in sub‐elite football teams experience that they are at high risk of sustaining groin injuries and believe that the Adductor Strengthening Programme will be effective in reducing the risk of groin problems, suggesting that there is fertile ground for implementation. The single‐exercise approach was an important facilitator for the successful implementation, and the majority of the players spent <5 minutes to complete the program. However, in future dissemination the players’ reluctant motivation for maintaining the exercise protocol may be considered a potential barrier to implementation that should be addressed.

## CONFLICT OF INTEREST

None declared.

## AUTHOR CONTRIBUTIONS

JH, RB, EGW, and TEA were involved in the design of the study and the data collection. The authors jointly interpreted the data and wrote the paper.

## ETHICAL APPROVAL

This study has been approved by the South‐Eastern Norway Regional Committee for Medical Research Ethics (2015/1922/REK) and the Norwegian Data Inspectorate (45388/3/LT/LR).
