# Supplementary material for: Implementation of the Adductor Strengthening Programme: Players primed for adoption but reluctant to maintain — A cross‐sectional study
Source: Scand J Med Sci Sports. 2019 May 23;29(8):1092–100. doi: 10.1111/sms.13444 (PMC6851742; doi:10.1111/sms.13444)
Supplement: Supplementary file 1 [file SMS-29-1092-s001.docx]

| *Questions asked to all players*  **To what extent do you think footballers are exposed to groin injuries?** | |
| --- | --- |
| □ Highly |  |
| □ Moderately |  |
| □ Low |  |
| □ Don`t know |  |
| **To what extent do you think footballers need to prevent groin injuries?**  □ Highly  □ Moderately  □ Low  □ Don`t know |  |
| **What do you think are the most common causes of groin injuries among footballers? (Multiple answers possible)** |  |
| □ Too little training  □ Too much training  □ Too many matches  □ Hard tackles  □ Low muscle strength  □ Reduced mobility  □ Reduced recovery time between matches  □ Artificial turf  □ Other |  |

| **Is it more important to use the training time to play football than to conduct injury prevention?** |
| --- |
| □ Fully agree |
| □ Agree |
| □ Not sure |
| □ Disagree  □ Totally disagree  □ Don`t know  **The motivation of the coach affects the players motivation to conduct prevention exercises?**  □ Fully agree  □ Agree  □ Not sure  □ Disagree  □ Totally disagree  □ Don`t know |

|  | **Very positive** | **Positive** | **Neutral** | **Negative** | **Very negative** | **Don`t know** |  |
| --- | --- | --- | --- | --- | --- | --- | --- |

**How do you perceive the general
attitude to preventive measures in
the following group in your club?**

Coaches □ □ □ □ □ □

Medical teams □ □ □ □ □ □

Players □ □ □ □ □ □

Administration □ □ □ □ □ □

*Questions asked to players in the intervention group*

**Are you familiar with the Adductor Strengthening Programme intended to prevent groin injuries?**

□ Yes

□ No

□ Don`t know

**Do you believe that the Adductor Strengthening Programme can reduce groin injuries?**

□ Yes, definitely

□ Yes, somewhat

□ No, I don`t think so

□ Don`t know

**Which players have primarily conducted the programme?**

□ All or most players

□ Players with groin problems

□ Players with previous groin problems

□ No players

□ Don`t know

**How has the execution of the programme been organized?**

□ When the players wanted, but not connected to organized training

□ When the players wanted, but connected to organized training (before or after training)

□ Together as a team connected to organized training

□ Don`t know

**Have you conducted the programme with the recommended frequency?**

□ More often

□ As recommended

□ Less often

□ Don`t know

**How much time did you spend conducting the Adductor Strengthening Programme?**

□ 0-5 min

□ 5-10 min

□ 10-15 min

□ Don`t know

**Will you use the programme after the current season?**

□ Yes, definitely

□ Yes, but not as frequency as this season

□ No

□ Don`t know

**Do you think the motivation to perform the Adductor Strengthening Programme would have been greater if the exercise was not a partner exercise?**

□ Yes, it is better to train alone

□ It does not matter

□ No, it is better to train with a team-mate

□ Don`t know

**Do you think the motivation to perform the programme would have been greater if it had contained several exercises?**

□ Yes, the more exercise the better

□ No, one exercise is sufficient

□ Don`t know

**How would the motivation to perform the programme have changed if it had taken less time?**

□ It would have increased

□ It would have decreased

□ Don`t know

**How would the motivation to perform the programme have changed if it had taken more time?**

□ It would have increased

□ It would have decreased

□ Don`t know

| **Are the following staff members familiar with the Adductor Strengthening Programme?** | **Yes** | **No** | **Don`t know** |  |  |
| --- | --- | --- | --- | --- | --- |
| Head and assistant coach | □ | □ | □ |  |  |
| Medical team | □ | □ | □ |  |  |
| Other coaches (fitness coach, goalkeeper trainer etc.) | □ | □ | □ |  |  |
|  |  |  |  |  |  |
| **How do you perceive the attitudes to the Adductor Strengthening Programme in the following groups?** | **Positive** | **Neutral** | **Negative** | **Don`t know** |  |
| Head and assistant coach | □ | □ | □ | □ |  |
| Medical team | □ | □ | □ | □ |  |
| Players | □ | □ | □ | □ |  |
| Administration | □ | □ | □ | □ |  |
|  |  |  |  |  |  |
| **Who has mainly initiated the programme? Rate from 1 to 3, where 1 is the one who has initiated it the most.** | **Most** | **Second most** | **Third** |  |  |
| Head coach | □ | □ | □ |  |  |
| Assistant coach | □ | □ | □ |  |  |
| Fitness coach | □ | □ | □ |  |  |
| Health professional | □ | □ | □ |  |  |
| Team captain | □ | □ | □ |  |  |
| Another player of the team | □ | □ | □ |  |  |
| The players of the team | □ | □ | □ |  |  |
| Don`t know | □ | □ | □ |  |  |

| **Who has mainly been responsible for the quality of the Adductor Strengthening Programme? Rate from 1 to 3, where 1 is the one who had most.** | **Most** | **Second most** | **Third** |
| --- | --- | --- | --- |
| Head coach | □ | □ | □ |
| Assistant coach | □ | □ | □ |
| Fitness coach | □ | □ | □ |
| Health professional | □ | □ | □ |
| Team captain | □ | □ | □ |
| Another player of the team | □ | □ | □ |
| The players of the team | □ | □ | □ |
| Don`t know | □ | □ | □ |

*Questions asked to players in the control group*

**Are you familiar with the content of the exercise program that players in the intervention group have conducted through the current season?**

□ Yes

□ No

□ Don`t know

**Have you conducted the preventive exercise program or other exercises to prevent groin problems during the current season?**

□ Yes

□ No

□ Don`t know
